# Genetic Analysis of High Protein Content in ‘AC Proteus’ Related Soybean Populations Using SSR, SNP, DArT and DArTseq Markers

**DOI:** 10.1038/s41598-019-55862-9

**Published:** 2019-12-23

**Authors:** Bahram Samanfar, Elroy R. Cober, Martin Charette, Le Hoa Tan, Wubishet A. Bekele, Malcolm J. Morrison, Andrzej Kilian, François Belzile, Stephen J. Molnar

**Affiliations:** 1 Agriculture and Agri-Food Canada, Ottawa Research and Development Centre, Ottawa, ON Canada; 2 Department of Biology and Ottawa Institute of Systems Biology, Carleton University, Ottawa, ON Canada; 3 Diversity Arrays Technology Pty Ltd, University of Canberra, Monana St., Canberra ACT, Australia; 4 Département de Phytologie and Institut de Biologie Intégrative et des Systèmes, Université Laval, Québec City, QC Canada

**Keywords:** Plant breeding, Plant molecular biology

## Abstract

Key message: Several AC Proteus derived genomic regions (QTLs, SNPs) have been identified which may prove useful for further development of high yielding high protein cultivars and allele-specific marker developments. High seed protein content is a trait which is typically difficult to introgress into soybean without an accompanying reduction in seed yield. In a previous study, ‘AC Proteus’ was used as a high protein source and was found to produce populations that did not exhibit the typical association between high protein and low yield. Five high x low protein RIL populations and a high x high protein RIL population were evaluated by either quantitative trait locus (QTL) analysis or bulk segregant analyses (BSA) following phenotyping in the field. QTL analysis in one population using SSR, DArT and DArTseq markers found two QTLs for seed protein content on chromosomes 15 and 20. The BSA analyses suggested multiple genomic regions are involved with high protein content across the five populations, including the two previously mentioned QTLs. In an alternative approach to identify high protein genes, pedigree analysis identified SNPs for which the allele associated with high protein was retained in seven high protein descendants of AC Proteus on chromosomes 2, 17 and 18. Aside from the two identified QTLs (five genomic regions in total considering the two with highly elevated test statistic, but below the statistical threshold and the one with epistatic interactions) which were some distance from Meta-QTL regions and which were also supported by our BSA analysis within five populations. These high protein regions may prove useful for further development of high yielding high protein cultivars.

## Introduction

Seed protein content is an important economic factor since whole or crushed soybeans are used as animal feed and also for human consumption. Through plant breeding, high seed protein alleles have been selected within cultivated soybean (*Glycine max* (L.) Merr.) germplasm or through introgression from wild *G. soja* germplasm^1,2^. Notably, the high seed protein cultivar AC Proteus^3^ was developed for short season Canadian conditions and it has become the parent of numerous current varieties with high seed protein^4^. Previous work has indicated that populations developed from AC Proteus may not exhibit the typical inverse relationship between seed yield and seed protein^5^. These desirable attributes of AC Proteus have not yet been investigated using molecular genetic approaches.

Molecular markers in plant breeding have a broad scope of applications, including but not limited to, genotyping, germplasm characterization, genetic diversity studies, genetic mapping, and QTL analysis^6^. Molecular breeding employs a breeding procedure called Marker Assisted Selection (MAS) in which DNA marker detection and selection are incorporated into a traditional breeding program^7,8^.

Molecular markers have been used as an important set of tools in many field crop breeding programs due to their reproducibility in large quantities, their stability when exposed to environmental changes and their independence from any tissue or growth stage^9,10^. Single-nucleotide polymorphism (SNP) is the variation of a single nucleotide at a specific location on the genome among individuals^10^. SNPs are common in plant genomes appearing every 100–300 bp or less^6^ and about ninety percent of human sequence variations are due to SNPs^11^. Therefore, SNPs used as DNA markers are very useful due to their abundance, stability, efficiency, ease in automation and lower assay cost^9,10,12^.

In this study we have included diversity arrays technology (DArT), and diversity arrays technology with next generation sequencing combined (DArTseq) markers for recombination mapping in soybean and also produced an integrated SSR, DArT^13^ and DArTseq^14^ marker-based recombination map for soybean^15,16^ to facilitate comparative mapping with the widely used soybean SSR composite map^17^ and other genomic studies. DArT marker genotyping has many advantages; particularly that it is a high throughput array-based system which has no prerequisite for genomic sequence information. DArT marker technology is now successfully deployed in a wide range of crop plants and was developed for soybean^15,16^. DArTseq markers are SNP-type markers detected on a DArT-type platform which takes advantage of the dramatic drop in the sequencing cost in the last decade and this enhanced technology has now largely replaced the original DArT. DArTseq does not depend on the availability of reference sequence for the genome (marker data extraction is “reference-free”), but enables immediate alignment of detected markers to the reference when it is available, which is the case for soybean. The present study was designed to investigate the genetics of high seed protein in AC Proteus using molecular genetic approaches by studying the high seed protein content loci in bi-parental populations and in AC Proteus-derived high protein cultivars.

## Materials and Methods

### Germplasm - Bi-parental population development and phenotyping

Three high protein parental lines were used in this study:

1. AC Proteus is an elite high protein cultivar adapted to early maturity zones in Ontario and Quebec^3,18^. The pedigree of AC Proteus is Merit/PI 153293/2/PI 189950/3/3*Maple Arrow^3^. Merit was developed at Agriculture Canada, Ottawa in 1960. PI 153293 was a high protein introduction from Belgium. PI 189950 was a very small seeded, high protein introduction from France (originally identified as *G. gracilis* now *G. max*).
2. X3144-48-1-B was developed from the cross AC Proteus/Maple Glen. Maple Glen is a high yielding cultivar^3^. X3144-48-1-B has the same pedigree as population X3585 used in a previous study of breeding for high protein^5^ but was independently developed.
3. X3145-B-B-3-15 has the pedigree BD22115/DW-8-3(X656-54)//CS-251-2(X1205-24-B-1)/3/Maple Glen, where BD is Amsoy/Portage//PI 438477, and DW is Renville/Capital(M387)//(M406)Harosoy/Norchief(M62-173)/3/USDA T106, *G. soja*, and CS is Hardome/PI 189950, *G. gracillis*//Merit/PI 153293/3/PI 438475.

Three low protein parental lines were used in this study:

1. Maple Arrow is the low protein recurrent parent used in the development of AC Proteus.
2. 9063^18^.
3. AC Brant^3^.

Five high x low seed protein and one high x high seed protein recombinant inbred line (RIL) populations were used in the present study:

1. XH939 is AC Proteus/Maple Arrow. This is an F6 derived RIL population. AC Proteus is a backcross two line derived from Maple Arrow and this cross is a backcross three population developed by Dr. Richard Buzzell at the Harrow Research and Development Centre of Agriculture and Agri-Food Canada.
2. X4049 is X3145-B-B-3-15/9063. This is an F5 derived RIL population.
3. X4050 is X3145-B-B-3-15/AC Brant. This is an F5 derived RIL population.
4. X4074 is X3144-48-1-B/9063. This is an F5 derived RIL population.
5. X4075 is X3144-48-1-B/AC Brant. This is an F5 derived RIL population.
6. X4038 is X3145-B-B-3-15/X3144-48-1-B. This is an F5 derived RIL population derived from a high protein x high protein cross.

Phenotyping of these populations was carried out at the Central Experimental Farm at Ottawa, Canada from 1997 to 2000. The X4050 population was also grown in 1999 at Exeter, Listowel, and Woodstock, ON and St-Cesaire, Ste-Rosalie, and Plessisville, QC. Population XH939 was only grown for three years (1998 to 2000) at Ottawa. Seed protein and oil content of field grown RIL populations were determined with infrared transmittance spectroscopy (Infratec 1241, FOSS) and expressed on a dry matter basis.

### DNA extraction

DNA was extracted from frozen leaves of plants grown in the greenhouse or the field using a modified urea extraction technique^19^.

### Markers, recombination mapping and QTL analysis

Previously designed soybean SSR primers^17^ were used in this study for DNA amplification. DArT and DArTseq marker analyses were performed as described elsewhere^13,15,16,20^. To assist with interpreting the recombination map, please note that typical nomenclature for microsatellite or Simple Sequence Repeat (SSR) markers is Satt100, for DArT markers it is soPb_100000 and for DArTseq markers it is 1000000. QTL analysis was performed with the software program MQTL^21,22^. Ten thousand permutations of the data were used to calculate the threshold for QTL detection. Regions with a test statistic above the threshold were considered a QTL. The major QTL was anchored and the map was re-scanned for regions that have additive or epistatic effects.

### AC Proteus genome-wide allele analysis

**Figure 1:**
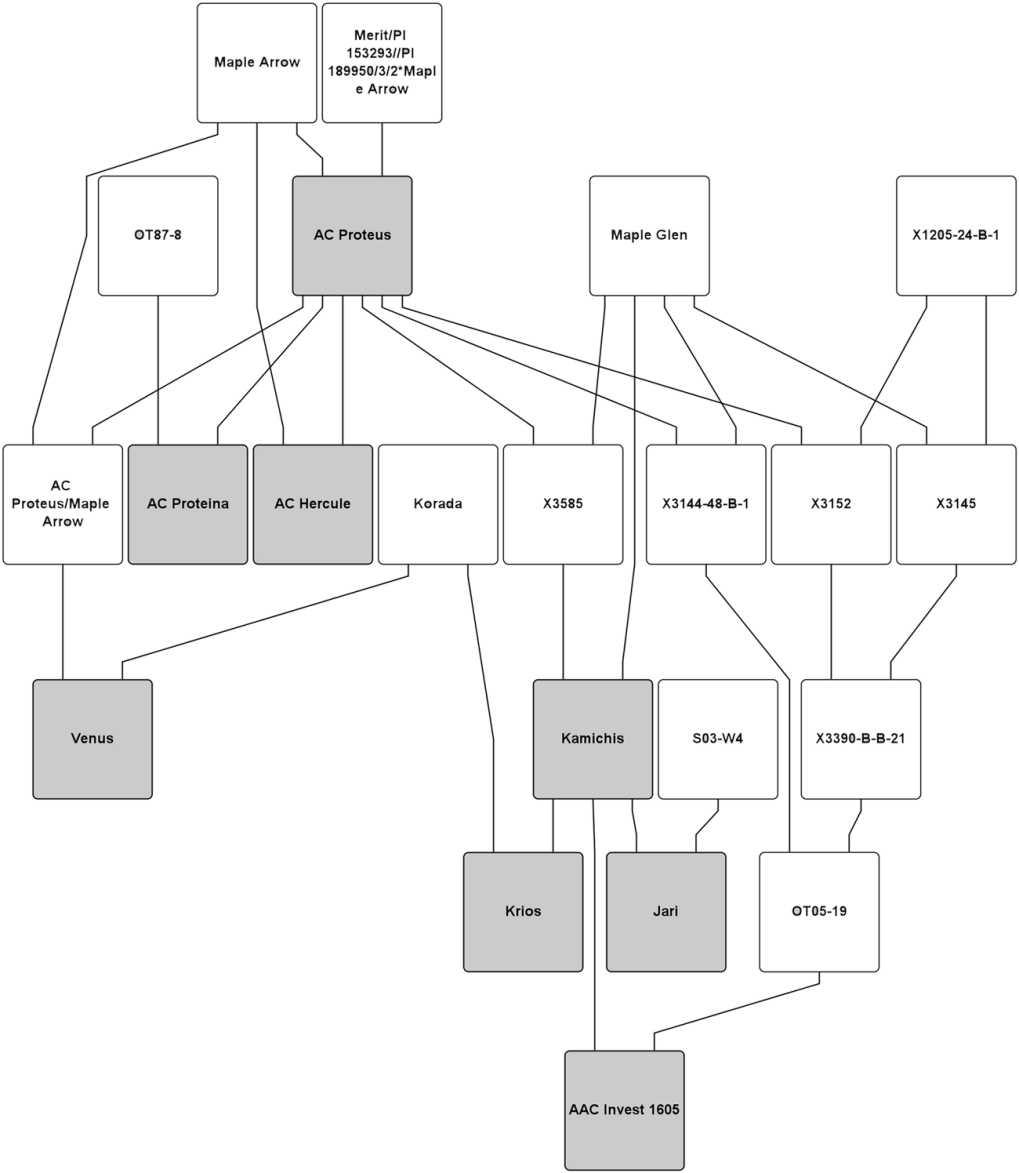
Pedigrees of high protein soybean AC Proteus and its high protein progeny. High protein cultivars used in the current SNP pedigree study are shown in grey.

A Genome by Sequencing (GBS) database of 155616 SNPs characterized across 300 Canadian soybean varieties^23,24^ was used as a source of genotype information for SNP haplotype analysis. Tassel 5 was used to sort the SNP data set for rare allele frequency analysis (AC Proteus rare allele frequency varies from 0.05–1.1% (ratio of 1–0.6, AC Proteus rare allele frequency in contrast to the other lines; 1 represents 100% match, while 0.6 represents 60% match) of the entire allelic frequency presented within the SNP panel) for AC Proteus (http://www.maizegenetics.net/tassel)^25,26^. Using the Canadian soybean collection of GBS-SNP data, AC Proteus alleles, at homozygous loci, were compared with seven AC Proteus derived high protein cultivars (AC Hercule, AC Proteina, Venus, Kamichis, Krios, AAC Invest 1605, Jari) and SNPs that were common across 66% of the derived high protein lines were identified; the first step was to identify AC Proteus alleles that were rare in the database. These SNPs were then compared to the low protein cultivars Maple Arrow, AC Brant (low protein cultivar), and Maple Glen (high yield cultivar) which were parents of populations. A second analysis was also carried out of those SNPs in which the criterion was that the AC Proteus allele was common across all AC Proteus derived high protein lines. Pedigree information of key cultivars used in this analysis are presented in Fig. 1, where the high protein cultivars are shown in grey. The pedigree graph was created using Helium software^27^.

## Results

### Protein content of parental germplasm

Values for seed protein and oil from trials at Ottawa were measured for several of the parental lines of the Ottawa derived RIL populations (Table 1). The high protein parents had about 48% seed protein while the low protein parents had about 40% seed protein

**Table 1:** Least square means for seed protein and oil of parental and check cultivars grown from 1998 to 2000 at Ottawa.

Genotype	n^a^	Protein (%)	Oil (%)
X3144-48-1-B	9	46.9	18.5
X3145-B-B-3-15	9	49.6	17.2
AC Proteina^b^	13	46.6	18.5
AC Brant	6	40.5	22.3
9063	13	39.8	22.2
S 00–66^c^	15	39.8	22.3
Korada^c^	15	41.7	20.8
OAC Bayfield^c^	11	40.6	22.0
Standard error		0.8	0.4

^a^Number of trials in which each line was grown. If a line was grown in every trial, n = 15, based on 3 years x 5 trials.

^b^Low protein check lines.

^c^High protein check lines.

**Figure 2:**
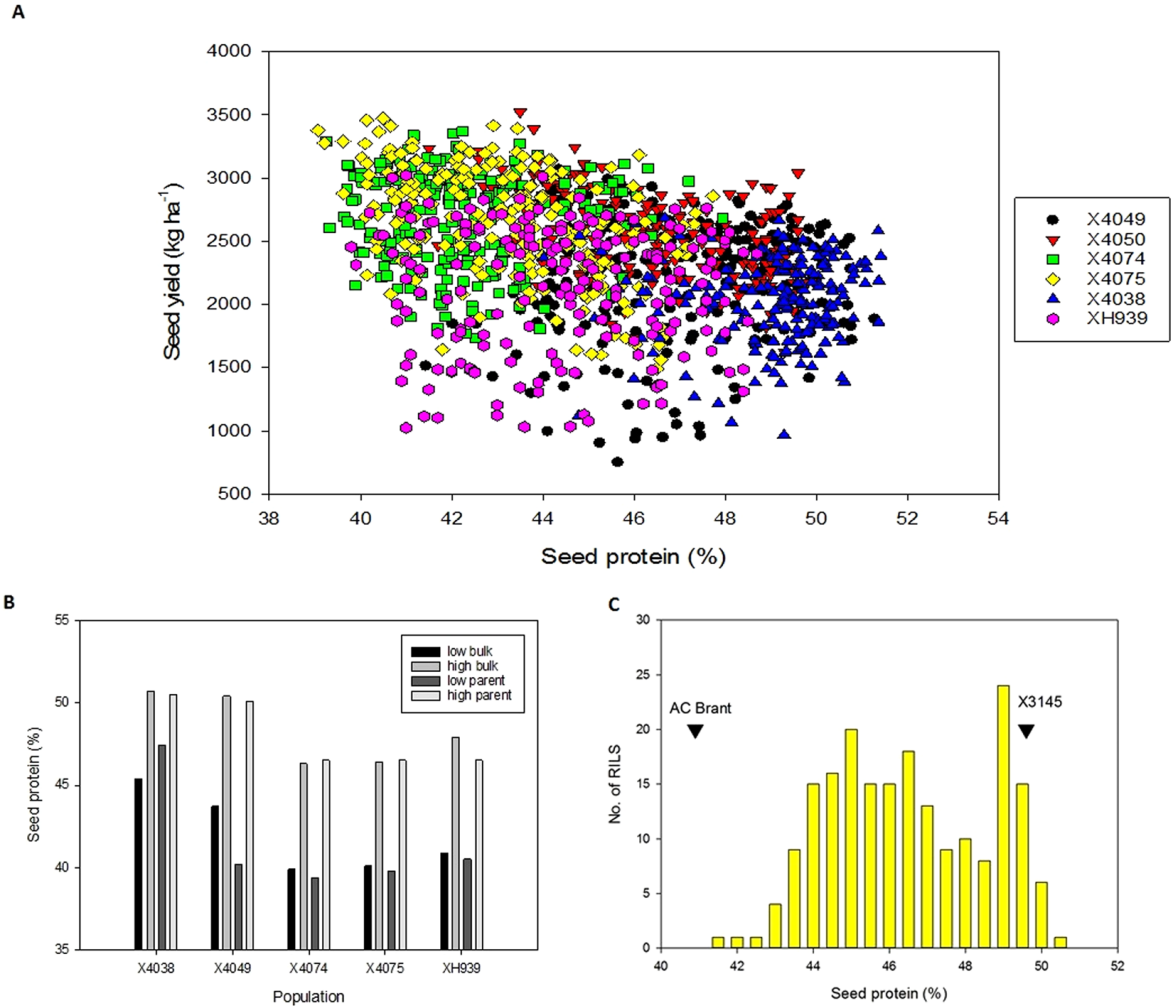
(**A**) Seed protein (%) versus seed yield (Kg ha^-1^) for all six populations. (**B**) Mean protein content of low and high protein bulks and parents for the X4038, X4049, X4074, X4075 and XH939 populations. (**C**) Seed protein histogram for RIL population X4050 and parents.

The six RIL populations showed variation for seed protein and seed yield (Fig. 2A). One population (X4050) was selected for detailed QTL analysis (Fig. 2C). This population was chosen because of the four Ottawa populations derived from high x low protein parents, the X4050 population’s frequency distribution for protein content most closely approximated a standard normal distribution. As a complementary cost-efficient strategy, high and low protein bulks from the other five populations (X4038, X4049, X4074, X4075 and XH939) were selectively genotyped (Fig. 2B).

### Recombination mapping with SSR, DArT and DArTseq markers

**Figure 3:**
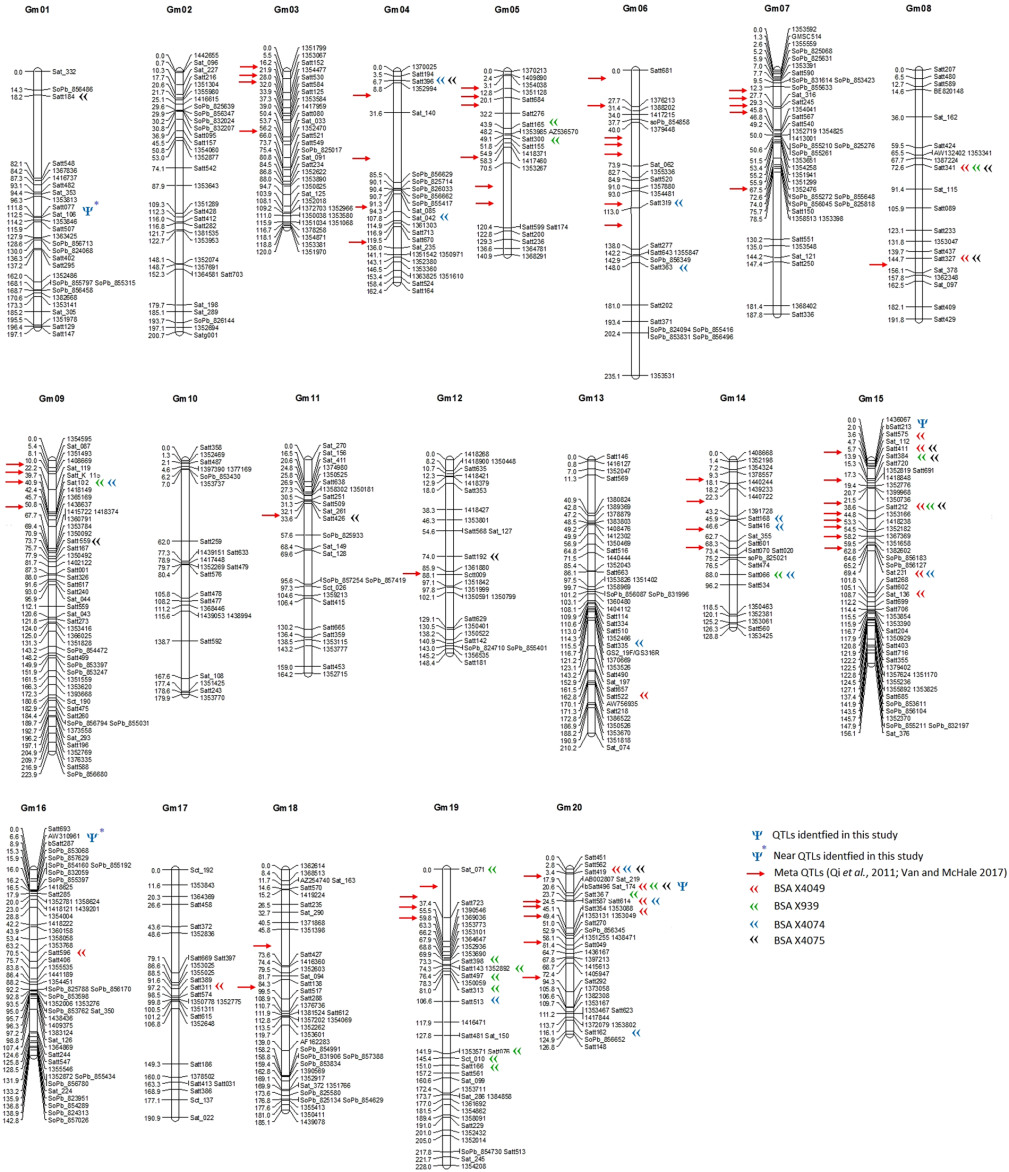
Recombination map for the X4050 RIL population. QTLs and near QTLs identified in X4050 and regions identified by BSA in the remaining populations (X4038, X4049, X4074, X4075 and XH939) have been added in this map. For comparison published protein Meta-QTLs^28,30^ are also shown.

In preparation for QTL analysis, a recombination map was developed in the X4050 RIL population (n = 100) using novel DArT and DArTseq markers as well as the widely used SSR markers. The resulting map (Fig. 3, Table 2) contains 264 SSR markers^17^, 83 DArT markers, and 297 DArTseq markers, for a total of 644 molecular markers. This is believed to be one of the very few soybean recombination maps with DArT and DArTseq markers co-mapped with SSR markers^15^, which facilitates comparative mapping between emerging DArT and DArTseq maps and the many published SSR based soybean maps and studies.

**Table 2:** Statistics of the recombination map for soybean population X4050.

Linkage Group	Chromosome	No. of mapped markers				Marker distance (cM)		
		SSR	DArT	DArTseq	Total	Average	Min	Max
D1a	Gm01	13	6	9	28	7.0	0.7	63.9
D1b	Gm02	14	13	5	32	6.3	0.3	27.4
N	Gm03	11	1	20	32	3.7	0.7	11.4
C1	Gm04	10	5	9	24	6.8	0.3	53.9
A1	Gm05	10	0	10	20	7.0	0.9	49.9
C2	Gm06	8	6	9	23	10.2	0.7	45
M	Gm07	11	12	16	39	4.8	0.6	51.7
A2	Gm08	17	0	4	21	9.1	1.7	23.5
K	Gm09	21	6	22	49	4.6	0.7	16.9
O	Gm10	11	1	12	24	7.5	0.7	55
B1	Gm11	15	3	8	26	6.3	0.4	26
H	Gm12	9	2	15	26	5.7	0.6	20.3
F	Gm13	16	2	23	41	5.1	0.7	29.6
B2	Gm14	10	1	12	23	5.6	0.1	20.9
E	Gm15	20	6	22	48	3.2	0.3	17.1
J	Gm16	11	16	21	48	2.9	0.1	17.2
D2	Gm17	15	0	9	24	7.9	0.7	42.5
G	Gm18	13	7	19	39	4.7	0.6	27.8
L	Gm19	17	1	19	37	6.2	0.9	37.4
I	Gm20	16	2	17	35	3.6	0.6	21.9

### QTL analysis for protein content in X4050

**Figure 4:**
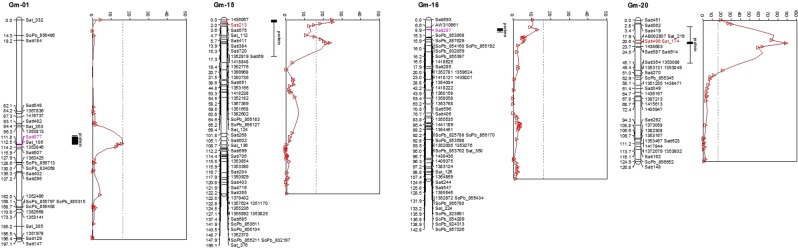
Scans of test statistic (composite interval mapping) for declaring a QTL (or near QTL) in X4050 for soybean protein content. SSR, DArT and DArTseq markers used for QTL analysis are a subset of those on the map in Fig. 3. The vertical line indicates the test statistic threshold for significance in declaring a QTL.

QTL analysis in population X4050 for protein content was performed using a map containing SSR, DArT and DArTseq markers (Figs. 3 and 4, supplementary file 1).

As presented in Fig. 4, two QTLs for seed protein content were detected, one on chromosome 20 (LG I, at SSR marker Satt496/Sat_174, explaining 60% of the population variation) and one on chromosome 15 (LG E, Satt213, 23%). In addition, there were two genomic regions with a highly elevated test statistic, but below the statistical threshold required to declare a QTL; one on chromosome 1 (LG D1a, Satt077, 14%), and the other one on chromosome 16 (LG J, Satt287, 13%). A region on chromosome 5 (LG A1, at DArTseq marker 1368291, 1%) was detected based on its epistatic interaction with the large QTL on chromosome 20 (data not presented).

### Bulk segregant analysis for protein content

An additional five populations were studied in an effort to validate QTLs found in the X4050 population, assess their applicability across germplasm, and perhaps detect additional relevant loci. Four of these populations (X4049, XH939, X4074, and X4075) were amenable to bulked segregant analysis (BSA) and therefore high and low protein bulks were selectively genotyped. Bulks were similarly genotyped in the fifth population (X4038), however, because it is a cross between two high protein parents, the results are more challenging to decipher. Therefore, classical BSA was not used in the X4038 population to discover high protein loci; however, the genotypes of the X4038 population bulks could be used to follow alleles at loci identified by BSA in the other four populations (Figs. 3 and 4).

Several genomic regions of interest were identified by comparison of the results obtained through QTL analysis and BSA (Fig. 3) (total of 37 locations identified by BSA analysis among the five populations). Among the four populations used for BSA, particular attention was given to positive BSA results with population XH939. That is because population XH939 (AC Proteus x Maple Arrow) is a “quasi-near isogenic population” since AC Proteus is a back cross two with Maple Arrow as the recurrent parent, with selection in each generation for high seed protein content, and XH939 is the third back cross to Maple Arrow. Thus, the high and low protein bulks derived from the XH939 population used for BSA should be highly specific for the genetic loci and/or alleles responsible for high seed protein in AC Proteus. Results from the present study were then compared to published results from GWAS, genome wide association study, analysis for high seed protein using some of the same germplasm^23,24^ as well as to published results for QTL analysis for high seed protein content (Soybase.org).

### Genome-wide approach to identifying AC Proteus rare alleles

A database of SNP genotypes of 300 Canadian soybean cultivars created by^23,24^ was used as a source for SNP haplotypes to investigate rare alleles in AC Proteus. Since there are a limited number of high protein lines in the Canadian SNP database, high protein alleles may appear rare but be present at higher frequency in the global germplasm and correspond to genomic regions previously reported in the high protein soybean literature.

For the initial broad analysis using the Canadian SNP database, we looked for rare AC Proteus alleles common across two-thirds or more of seven AC Proteus derived lines (AC Hercule, AC Proteina, Kamichis, Krios, Venus, Jari and ACC Invest 1605) but absent from the low protein parental lines (Maple Arrow, AC Brant and Maple Glen). AC Proteus descendants had been developed through up to three additional breeding cycles with continuous selection for high protein.

A total of 155,616 SNPs were screened for alleles present in AC Proteus but rare within the SNP database. This subset of SNPs (1,721) was further screened for those that contrasted between AC Proteus and its low protein recurrent parent Maple Arrow and additionally those where the AC Proteus allele was present with an allelic ratio of 0.66 or greater among the seven high protein derivatives of AC Proteus. Based on the selected ratio of 1–0.66, 0.05–1.1% of the alleles present within the SNP panel were selectively retained by AC Proteus and its derivatives. As shown in Supplementary file 2, the approximately 650 SNPs that met this set of criteria were sometimes in close physical proximity to each other and appear to define genomic blocks, which may represent haplotypes for high protein. Using linked SSR markers to bridge between the recombination map (Fig. 3) and genomic sequence map (Soybase, assembly 2.0) it was possible to demonstrate that five of the SNP blocks correspond to either QTLs identified in X4050 or positive genomic regions identified by BSA in the other four RIL populations (Table 3). Two of those five blocks also correspond to published Meta-QTL for protein content. An additional three blocks align with other published Meta-QTLs for protein content (Table 3). These correspondences help validate the results obtained by these three independent analytical methodologies (QTL, BSA, SNP based pedigree analysis) and support the hypothesis that these eight and possibly more genomic regions play a contributory but not necessarily essential role in the high protein and high yield phenotype of AC Proteus and derived breeding lines. It is also noteworthy that the blocks vary considerably in size. For example those in Table 3 vary from 150 kb to 11,000 kb, and the larger blocks may carry multiple genetic loci that have been retained through selection for high protein.

**Table 3:** Eight genomic blocks containing SNPs having high AC Proteus rare allele frequency (0.667 to 1.0) and their linked SSR loci.

Chromosome	Position	Linked SSR or DArT loci and corresponding QTL, BSA or Meta-QTL known loci
1	49056999–49869514	Satt077, linked to QTL identified in this study
4	2641058–2804682	Satt396, 2 BSA, and linked to Meta-QTL7
4	40051535–44359031	Sat_042, 1 BSA, and linked to Meta-QTLs 18 and 19
4	51139624–52083889	Sat_140, and linked to Meta-QTL8
7	7101188–15400818	Satt245 and Satt590, and linked to Meta-QTL mPO7–5 and mPO7-6
9	29841368–31206660	Satt326, and linked to Meta-QTL mPO9-4
15	16796344–27683694	Sat_136, Satt268, 1 BSA, and linked to Meta-QTL mPO15-3
16	2392186–2955745	Satt287 and SoPb_853068, linked to QTL identified in this study

Each block corresponds to a protein QTL identified in X4050, or a region of interest identified by BSA, or a published protein Meta-QTL (Supplementary file 2 and Fig. 3).

**Figure 5:**
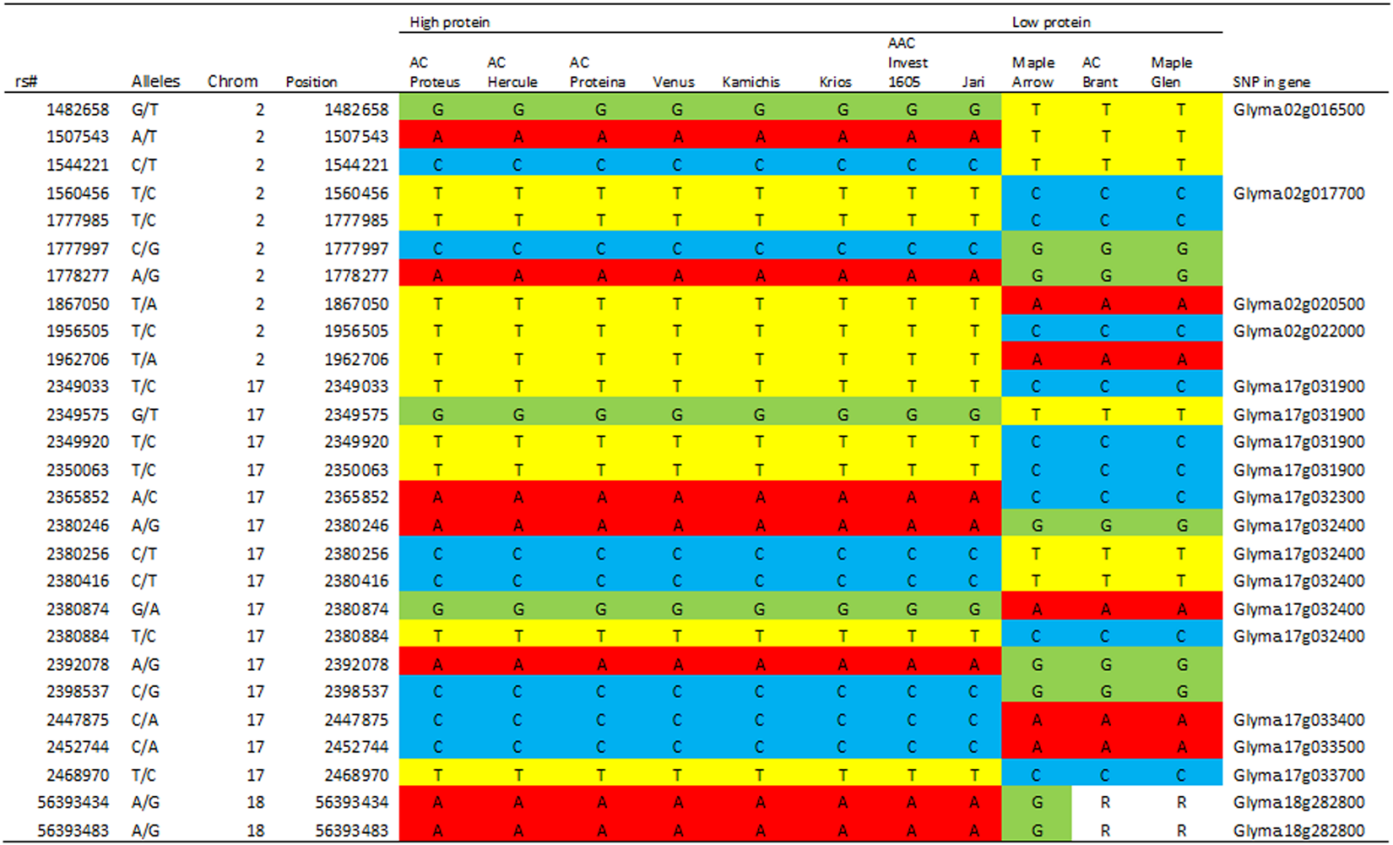
Genome-wide analysis of AC Proteus rare alleles, which were maintained across three cycles of breeding for high protein in all seven derived high protein soybean cultivars, and which contrast with Maple Arrow, the recurrent parent of AC Proteus. All the items included in Table 3, are excluded from Fig. 5.

In a second analysis of the SNP data, the same strategy, but employing more stringent screening criteria (allele frequency of 1.0), was used to search for essential and perhaps novel alleles responsible for the desirable high protein phenotype of AC Proteus and its descendants. AC Proteus SNP alleles (not shared by Maple Arrow) which are rare in the Canadian germplasm but retained in all seven AC Proteus derived cultivars were identified. Those which were not already reported in Table 3 are shown in Fig. 5. These criteria were met by 7 blocks (11 genes) of SNPs. These blocks are identified on chromosomes 2, 17 and 18. Such putatively novel regions that are perfectly conserved through multiple breeding cycles may carry genes having important high protein alleles derived from AC Proteus. Also shown in Fig. 5 are those SNPs which are located within genes, however none are implicated as candidate genes by the current analyses.

## Discussion

Taken together, the five genomic regions identified in this study account for 70% of the phenotypic variation for seed protein in this population. Major QTLs for protein content have been identified on chromosome 20; this region corresponds to the most frequently reported protein content QTL in the literature and to the protein content Meta-QTL #18^28^. However, the QTL identified on chromosome 20 is distant (~4–6 cM, map unit) from the reported Meta-QTLs, and also supported by BSA analysis in three different population (X4049, X4074, and XH939), and can thus be considered as a new QTL. The second QTL for protein content is at Satt213, on chromosome15; Satt213 is distant from the closest protein content Meta-QTL and likely an independent locus, and supported by BSA analysis at close proximity. However, Satt213 is tightly linked to the QTL seed protein content 1–5 (the peak marker is RFLP pSAC-7a aka pSAC7_1), identified in the A81356022 (*G. max*) x PI468916 (*G. soja*) population^29^ and reported in SoyBase.

The major protein content QTL on chromosome 20 was detected by BSA at Satt496 in three of the four populations investigated in this study, and by BSA at the adjacent marker Satt587 in the fourth population. Additional positive BSA results at flanking markers support the hypothesis that the identified locus on chromosome 20 is likely the major locus for protein content in all five populations. The related shoulder peak (significant peaks close to the major peak) at Satt419 and Satt562 on chromosome 20 was also detected by BSA in three of the four populations.

The second protein content QTL detected in population X4050 was at Satt213 on chromosome 15. BSA at the flanking marker Satt411 was positive for two of the four populations investigated in this study (X4049, and X4075), while the high protein parent’s allele was fixed in the other two populations. Note that this is consistent with the hypothesis that this locus is very important (significant) for achieving high protein content in all five populations. BSA identified other loci potentially important for protein content which were not detected by QTL analysis for protein content in population X4050. Further along on chromosome 15, Satt212 was positively identified in three populations and fixed for the high protein allele in the fourth. On chromosome 8, BSA gave a positive result for Satt341 in three populations and the fourth population was fixed for the high protein parent’s allele. Also, on chromosome 8, BSA gave a positive result for two populations at Satt327. Since the two markers are approximately 30 cM apart, they may well represent different loci. Satt341 and Satt327 span a genomic region with numerous seed composition QTL reported on SoyBase.

BSA gave positive results in two of the four populations at several additional loci. The first was at Satt066 on chromosome 14, which is tightly linked to the protein content Meta-QTL6^28^. At Satt396 on chromosome 4, which is linked to protein content Meta-QTL7, a third population was fixed for the high protein parent’s allele. On chromosome 15, BSA gave positive results for Satt384 in two populations while a third was fixed for the high protein allele. The Satt384 locus is linked to protein content Meta-QTL mPO15–2^30^. Also, on chromosome 15, Satt231 was highlighted by BSA and is linked to protein content Meta-QTL14^28^. In all four cases, linkage to a Meta-QTL would appear to validate the identification of these four loci by BSA and suggest that they contribute to achieving high protein content in this germplasm.

A positive BSA result in only one of the four populations might well be a false positive. However, it is worth noting that in the cases of Satt192 on chromosome 12 and Satt559 on chromosome 9, the other three populations were fixed for the high protein parent’s allele. Additionally, at Satt319 on chromosome 6, two of the three other populations were fixed for the high protein parent’s allele and the Satt319 locus is tightly linked to Meta-QTL11^28^.

A recent study^31^ using high protein parents AC Proteus and AC Proteina did not find protein QTLs in the AC Proteus population but did find QTLs on chromosome 15 and 20 in the commonly reported regions on the AC Proteus-derived AC Proteina population.

As presented in Table 3 and 4, AC Proteus, and derived high protein progeny, carry rare alleles in comparison to Canadian low protein germplasm but many of these regions are commonly identified in the high protein literature. Some novel regions were identified; none of the genes identified in Fig. 5 have Meta-QTL in close proximity except for Glyma.15g197800. To facilitate comparison of our SNP allele data with our QTL and BSA data, we have searched the soybean genomic sequence physical map near the AC Proteus rare alleles (SNPs) to identify the closest SSR marker (Supplementary files 2 and Fig. 3). These data are consistent with our hypothesis that AC Proteus may carry novel high protein alleles.

In summary, we developed a recombination map which integrates DArT and DArTseq markers with the widely used SSR markers. QTL analysis and bulk segregant analysis identified QTLs for high protein in our populations which correspond to important QTLs in previous research and supported with Meta-QTL analyses. We identified two QTLs for seed protein content on chromosomes 15 and 20 (five genomic regions in total considering the two with highly elevated test statistic, but below the statistical threshold and the one with epistatic interactions) which have not been included in Meta-QTL regions. It is worth mentioning, among all the regions identified by BSA in this study (Fig. 3 and Table 3), those located on chromosomes 1, 8, 9, 14, 16, 17, 19, and 20 are considered novel (identified in this study and no reported Meta-QTLs located in close proximity). We further identified regions on chromosomes 2, 17 and 18 which were maintained in high protein cultivars derived from AC Proteus over multiple breeding cycles. These high protein regions may prove useful for further development of high yielding high protein cultivars.

## Supplementary information

Supplementary Dataset 1

Supplementary Dataset 2
